# Enhancing Mechanical Properties and Microstructures of Mass-Manufactured Sand Concrete by Incorporating Granite Powder

**DOI:** 10.3390/ma17102234

**Published:** 2024-05-09

**Authors:** Jian Huang, Guangfeng Xu, Shujie Chen, Demei Yu, Tengfei Fu, Chao Feng, Yulin Wang

**Affiliations:** 1 The Fifth Construction Co.Ltd. of CCCC Fourth Harbor Engineering Co., Ltd., Fuzhou 350008, China; swordy-hj@163.com; 2 College of Transportation and Civil Engineering, Fujian Agriculture and Forestry University, Fuzhou 350108, Chinayudemei0826@fafu.edu.cn (D.Y.); 3 CSCEC Strait Construction and Development Co., Ltd., Fuzhou 350015, China; 4 School of Architecture and Civil Engineering, Wuyi University, Wuyishan 354300, China; 5 Engineering Research Center of Prevention and Control of Geological Disasters in the Mountainous Areas of Northern Fujian, Fujian Province University, Wuyishan 354300, China

**Keywords:** manufactured sand concrete, mechanical properties, heat of hydration, pore distribution, microstructure

## Abstract

The production of manufactured sand and stone processing can cause dust pollution due to the generation of a significant amount of stone powder. This dust (mainly granite powder) was collected and incorporated as a cement replacement into mass-manufactured sand concrete in order to enhance the mechanical properties and microstructures. The heat of the hydration was measured by adding the granite powder into the cementitious material system. The mechanical properties, autogenous shrinkage, and pore structures of the concrete were tested. The results showed that the mechanical strength of the concrete increased first and then decreased with the increase in granite powder content. By replacing the 5% cement with the granite powder, the 28 d compressive and flexural strength increased by 17.6% and 20.9%, respectively. The autogenous shrinkage was mitigated by the incorporation of the 10% granite powder and decreased by 19.7%. The mechanism of the granite powder in the concrete was studied by X-ray diffraction (XRD), scanning electron microscopy (SEM), and mercury intrusion porosimetry (MIP). The porosity decreased significantly within the 10% granite powder. A microstructure analysis did not reveal a change in the type of hydration products but rather that the granite powder played a role in the microcrystalline nucleation during the hydration process.

## 1. Introduction

Manufactured sand has become the focus of research and applications due to the shortage of natural sand in order to achieve low-carbon, green, and sustainable development in the construction industry. A large amount of stone powder is produced as a consequence of this process, thus polluting the air and rivers. The resource utilization of stone powder is an effective way to solve this problem [1,2]. Stone powder is used as a mineral admixture to replace part of the cement, and it will affect the performance of the concrete through a filling effect in cement-based materials [3,4]. The stone powder will accelerate the cement hydration in cement paste under the condition of a constant water–cement ratio through the dilution effect of the cement paste. This is beneficial to improve the early strength of cement concrete [5,6].

The use of granite powder to replace part of the cement can significantly improve the carbonization and durability of the cement mortar [7]. Jain et al. found that replacing sand and cement with 30% granite powder and 15% waste glass powder can improve the durability of the concrete [8]. Singh et al. found that the mechanical strength of concrete was significantly improved by adding 10% marble and 25% granite powder [9]. In addition, Singh et al. pointed out that 30% fine sand replaced by fine granite solid waste can produce concrete with the optimum compressive strength [10]. Shwetha et al. considered the feasibility of using granite dust and debris to improve the tensile and bending strength of concrete [11]. Other research found that the introduction of stone powder into Portland cement systems was beneficial in improving the volume stability of the mortar and concrete [12]. Chouhan et al. [13] provided a comprehensive review on the influence of various shapes and sizes of stone waste as partial replacements of cement and sand regarding the workability, mechanical, and durability properties of mortar mixes and concrete. These studies suggest that the dimensional stone waste enhanced the bond properties and the durability characteristics [13]. Additional details can also be found in the literature [13]. Mashaly demonstrated that up to a 20% granite sludge replacement of cement could enhance the freeze and thaw resistance, abrasion, and sulfate attack, with negligible decreases in the physical and mechanical properties [14]. In other studies, the addition of stone powder was found to reduce the early shrinkage of cement-based materials but increase the later shrinkage [15,16].

The granite powder mainly improved the microstructure and properties of the concrete via the filling effect (referring to the physical presence of fine mineral additions in cement to enable denser packing) and nucleation effect (referring to the surface of the mineral filler providing nucleation sites for C–S–H formation). The improvement generally increased with the replacement of the granite powder. Xiong et al. found that tobermorite was generated in the slurry of granite–stone powder composite cementitious material by studying the effects of different-lithology stone powders on the hydration products of cement-based materials [17]. Li et al. found that the concrete pores first increased and then decreased with the increase in the granite powder content [18].

Granite powder can improve the mechanical strength and elastic modulus of concrete with manufactured sand in later ages as a supplementary cementitious material replacing fly ash with different proportions [19]. The granite powder was beneficial regarding the high-strength concrete durability with manufactured sand within a certain range of replacements. However, too-high values of fine powder could inhibit the hydration of the cement and then adversely affect the durability of the resulting concrete [20]. Shen et al. found that the particle shape and surface texture of manufactured sand had less influence on the performance of the concrete than that of stone powder [21].

At present, there are few studies on the effect of granite powder on the performance and mechanism of mass concrete composed of manufactured sand. This work incorporated granite powder into mass concrete in the hope of reducing the heat generation due to hydration and increasing the utilization of solid waste materials. The working mechanism of the granite powder was analyzed via XRD and SEM. The hydration behavior of the cementitious material was studied through isothermal calorimetry. The mechanical properties and early shrinkage properties of the granite powder mass concrete were also evaluated.

## 2. Materials and Methods

### 2.1. Materials and Mixture Proportion

**Figure 1:**
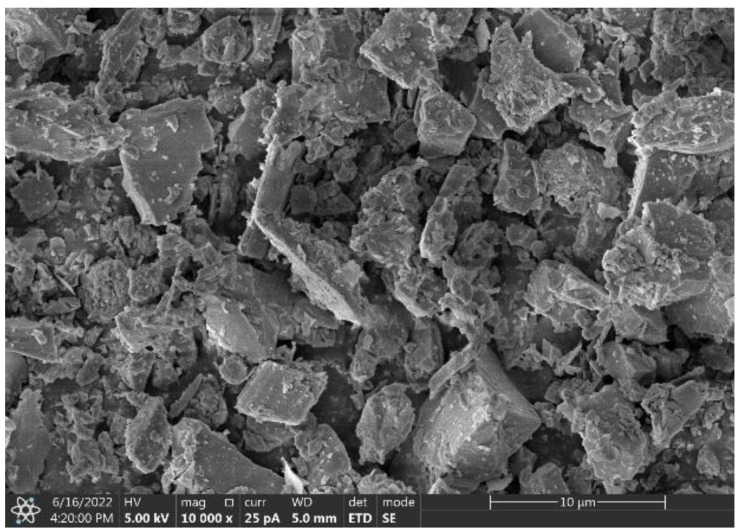
SEM image of granite powder (mag. ×10,000).

**Figure 2:**
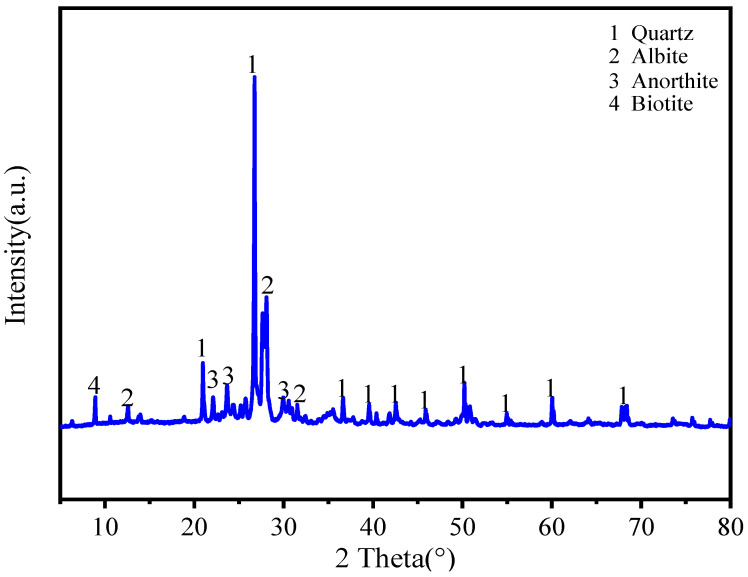
XRD pattern of granite powder.

The local P.O 42.5 cement (Lianshi Cement, Fuzhou, China) was used in this work. Granite powder (GP) was obtained from a local stone processing mill. Then, it was sieved through a 0.075 mm sieve. The specific surface area of the resulting powder is 3.075 m^2^/g with a density of 2.18 g/cm^3^. The chemical composition of the cement and granite powder from XRF analysis are shown in Table 1, and the SEM image and XRD diffractogram of the granite powder are presented in Figure 1 and Figure 2.

**Figure 3:**
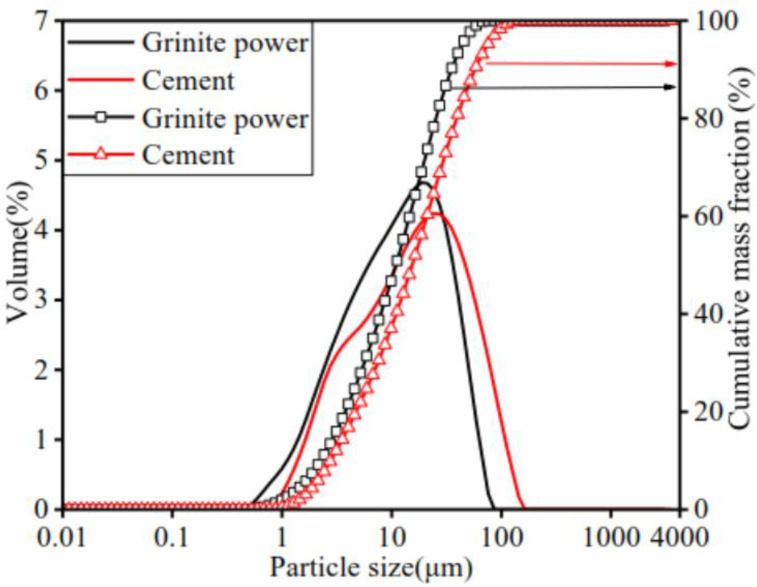
Particle distributions of cement and granite powder. (Cumulative mass fraction follows arrow to the right axis).

The granite powder particles have rough surfaces showing irregular granular shapes (Figure 1), and it is observed that the granite powder is in the form of quartz, albite, anorthite, and biotite (Figure 2). Figure 3 shows the particle distributions of cement and granite powder, showing that the sieved granite powder is finer than cement. The fineness modulus of the river sand was 2.82. The coarse aggregate of basalt was used with a gradation of 5–25 mm, and the apparent density was 2999 kg/m^3^. The mixing water was clean tap water. The fine aggregate used was combined using manufactured sand (MS) and natural fine sand (FS) according to a ratio of 0.64:0.36. The apparent density was 2596 kg/m^3^. Gradation of the fine aggregate is shown in Table 2.

Mixture proportions of mass-manufactured sand concrete with granite powder and fly ash are presented in Table 3. This particular mixture design was used for the Fuzhou subway roof, which is a 2 m deep reinforced concrete slab, to support the superstructures.

### 2.2. Experimental Methods

#### 2.2.1. Mechanical Properties Testing

Compressive strength of the concrete specimens was tested by compression testing machine (YAW4206, Shanghai, China). Flexural strength of the specimens was tested by an electro-hydraulic servo universal testing machine (HUT605A, Wan Testing Equipment, Shenzhen, China). Three specimens of 100 mm × 100 mm × 100 mm were tested based on China National Standard GB/T 50081-2019 [22]. Three 100 mm × 100 mm × 400 mm specimens were evaluated according to ASTM C78 [23]. All samples were cured in a standard condition of 20 ± 3 °C and 95 ± 5% RH.

#### 2.2.2. Heat of Hydration Measurement 

The hydration heat test was performed on a TAM Air isothermal calorimeter (TAM Air, TA Instruments, New Castle, DE, USA). The hydration exothermic rate and cumulative exothermic amount of different cementitious systems were measured according to ASTM C186-2017 [24]. The water–binder ratio was 0.38. For each mixture, the result was averaged between two specimens.

#### 2.2.3. Autogenous Shrinkage Measurement

The autogenous shrinkage of concrete was carried out according to ASTM C1698-2019 [25]. The prepared concrete was poured into a 420 mm (Φ70 mm) corrugated tube, placed horizontally on a steel frame. The room temperature was controlled at (20 ± 2) °C, and the relative humidity was above 50%. The results were averaged between three specimens.

#### 2.2.4. Pore Structure Analysis

Three specimens of 20 mm × 20 mm × 20 mm were prepared. The samples were soaked in anhydrous ethanol to stop the hydration of cement and dried in a 60 ± 5 °C vacuum oven for 5 h, then cooled to room temperature. The pore structure of concrete was measured by Auto Pore IV 9500 mercury intrusion porosimeter (Norcross, GA, USA).

#### 2.2.5. Microstructure Characterization

Samples of approximately 5 mm × 5 mm were taken from the AGP0 and AGP10 28 d specimens and dried and sputtered with gold. Microscopic morphology was observed using an electron microscope of model Verios G4 UC (Thermo Fisher Scientific, Waltham, MA, USA).

Mineral composition of concrete was analyzed using a D8 Advance type X-ray diffractometer (Bruker, Billerica, MA, USA). The 28 d concrete specimens were ground until all the pieces broke down and sieved through 0.075 mm sieve. The samples were made into 10 mm × 10 mm filament specimens. The testing condition was at 2θ of 5–90° and the step of 12°/min.

## 3. Results

### 3.1. Mechanical Properties

#### 3.1.1. Compressive Strength

**Figure 4:**
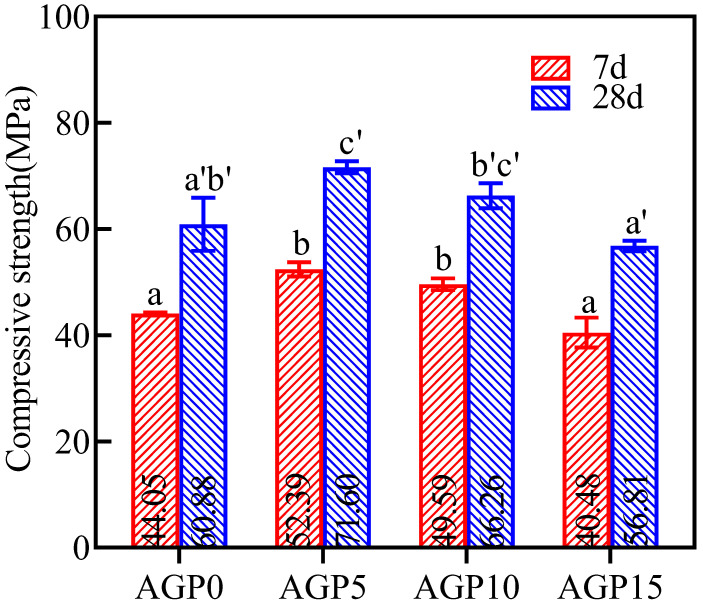
Compressive strength values of manufactured sand concrete with different stone powder content. Note: data were analyzed with one-way ANOVA. Significant differences exist between any two groups when they do not share a common letter over the columns (also applies for Figure 5).

The compressive strength of the manufactured sand concrete with different granite powder content is shown in Figure 4. The compressive strength of 7 d and 28 d peaked at the 5% granite powder content. Compared with the control group (AGP0), the compressive strength in the AGP5 and AGP10 groups at 7 d and 28 d increased significantly. AGP5 increased by 18.9% and 17.6%, and AGP10 increased by 12.5% and 8.8%, respectively. When the replacement exceeded 10%, the AGP15 group was 8.1% and 6.7% lower than the AGP0 group at 7 d and 28 d, respectively. Therefore, the high replacement of the granite powder could adversely affect the compressive strength.

#### 3.1.2. Flexural Strength

**Figure 5:**
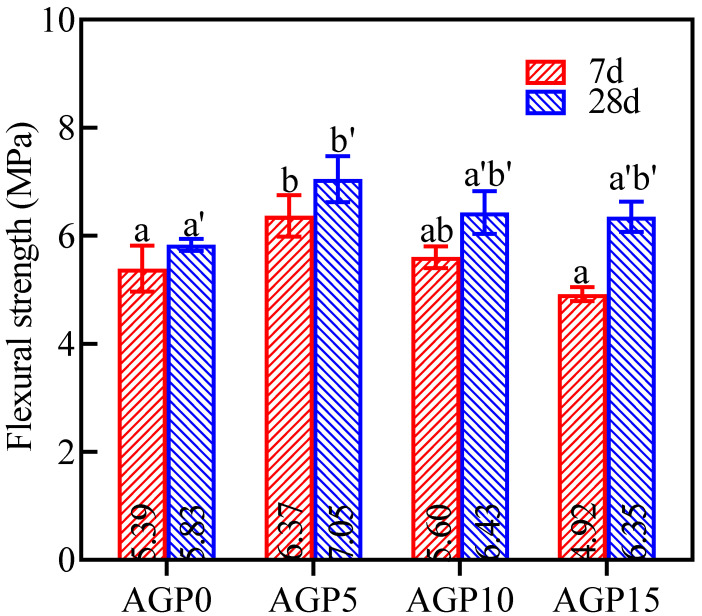
The flexural strength of manufactured sand concrete with different granite powder replacements.

The flexural strength of manufactured sand concrete with different granite powder content is shown in Figure 5. The flexural strength is similar to the compressive strength, and it increases first and then decreases with the increase in powder content. The 7 d and 28 d flexural strength of the concrete in AGP5 increased by 18.1% and 20.9% compared with the controls (AGP0), and AGP10 increased by 12.6% and 10.3%, respectively. However, a further increase in the replacement of the granite powder would not result in a significant increase in the flexural strength.

### 3.2. Heat of Hydration

**Figure 6:**
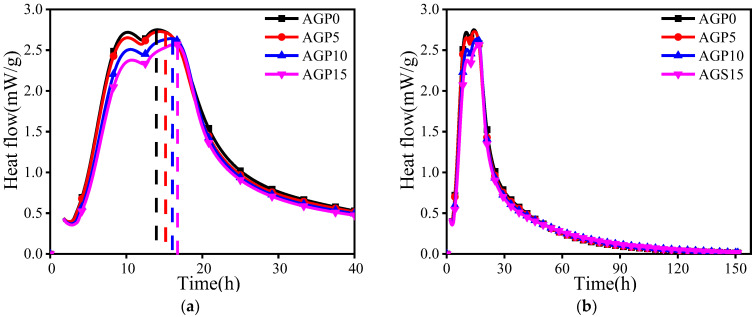
Effect of granite powder on hydration heat release rate of cement: (**a**) heat flow in the first 40 h (dashed line indicating corresponding time of heat flow peak); (**b**) heat flow within 150 h.

**Figure 7:**
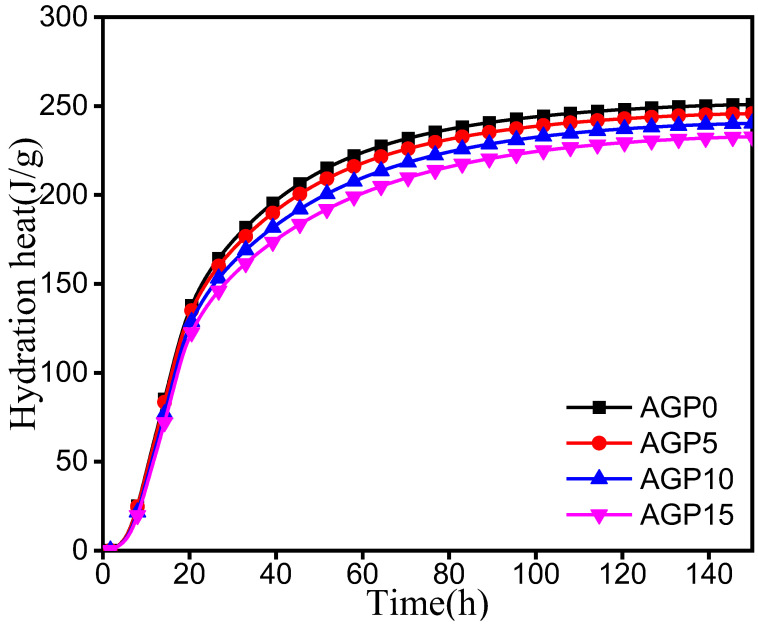
Cumulative heat release over time.

The effect of the granite powder on the heat release rate of the cement hydration is shown in Figure 6. The effect on the cumulative heat is shown in Table 4 and Figure 7. From Figure 6a, it can be seen that the peak of the heat evolution curve shifted to the right for the cement pastes blended with granite powder when compared to AGP0. Therefore, the granite powder has a slight retardation effect on the hydration of the cement. Table 4 and Figure 7 show that the granite powder can also decrease the hydration heat. The cumulative hydration heat values at 150 h for AGP5, AGP10, and AGP15 are 2.03%, 4.22%, and 7.23% lower than for AGP0. This is due to granite powder being considered inert material. Partially replacing the cement would inevitably reduce the total heat of the hydration in the system, which can be beneficial regarding the mass concrete casting by reducing the risk of early cracking due to the temperature gradient.

### 3.3. Autogenous Shrinkage

**Figure 8:**
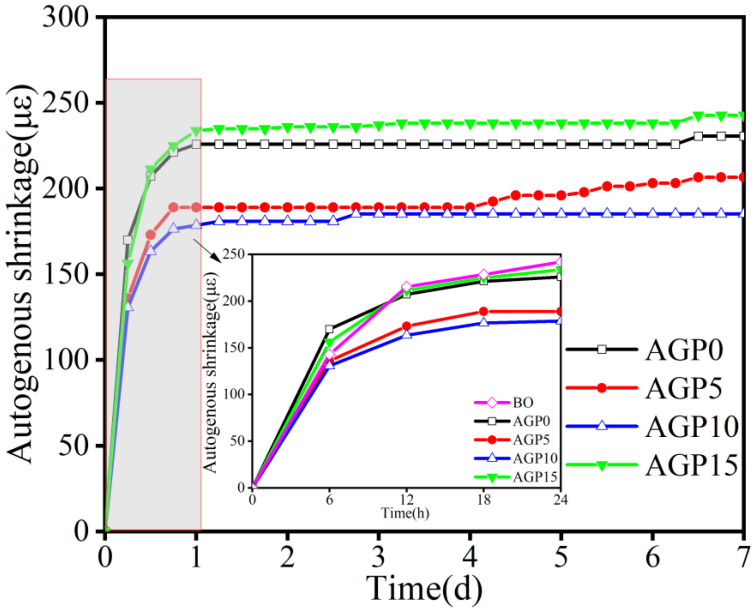
Effects of different amounts of stone powder substitution on autogenous shrinkage.

The effect of different granite powder content on the autogenous shrinkage is shown in Figure 8. It can be seen that the autogenous shrinkage of the concrete occurred mainly on the first day and did not change significantly in the next few days. The increase in the granite powder content had a greater effect on the autogenous shrinkage. The autogenous shrinkage rates of the AGP5 and AGP10 concrete groups were comparatively small. Compared with the AGP0 group, there were significant decreases of 10.4% and 19.7%, respectively. When the replacement reached 15%, the autogenous shrinkage rate of the concrete increased significantly. However, it should be noted that high powder content would create smaller pores (50 nm or less) so as to significantly increase the autogenous shrinkage.

### 3.4. Microstructure Analysis

#### 3.4.1. Pore Structure

**Figure 9:**
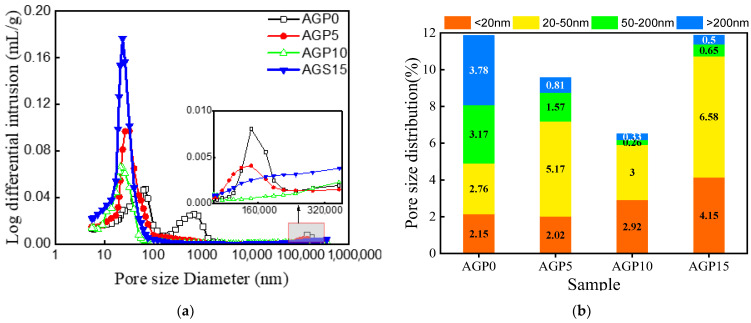
Pore-size distributions of concrete with different granite powder content: (**a**) differential intrusion; (**b**) pore-size distribution.

The results of the MIP test of the concrete pore structure are shown in Table 5 and Figure 9a. Based on the volume proportions of the pore structure distributions shown in Figure 9b, it can be seen that, when the granite powder replaces the cement in a certain range, the filling effect of the granite powder on the concrete is significant. With the increase in the granite powder content, the larger pores (≥50 nm) in the concrete are significantly reduced, the compactness of the concrete is improved, and the porosity is reduced. When the replacement amount of the granite powder continues to increase, the filling effect diminishes because the granite powder cannot fill the pores close to or smaller than its own size. This can also help to explain the test results of the mechanical properties.

#### 3.4.2. SEM

**Figure 10:**
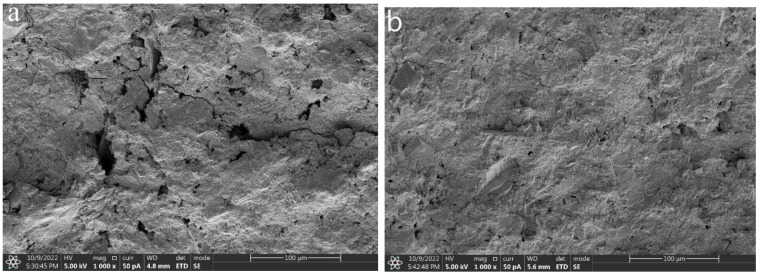
SEM images of 28 d hydration products (**a**) AGP0; (**b**) AGP10.

The SEM images of the 28 d hydration products of the two groups of AGP0 and AGP10 are shown in Figure 10. There was a significant difference with the addition of the granite powder. There were a large number of pores and micro-cracks between the hydration products, and the structure was not as dense. After the incorporation of the granite powder, it can be observed that the granite powder and the hydration products in AGP10 are cemented together (Figure 10b) with significantly decreased porosity compared to AGP0. Also, the number of micro-cracks is significantly reduced. This is likely due to the nucleation effect of the granite powder, forming a denser structure by combining the hydration products with stronger integrity.

#### 3.4.3. XRD

**Figure 11:**
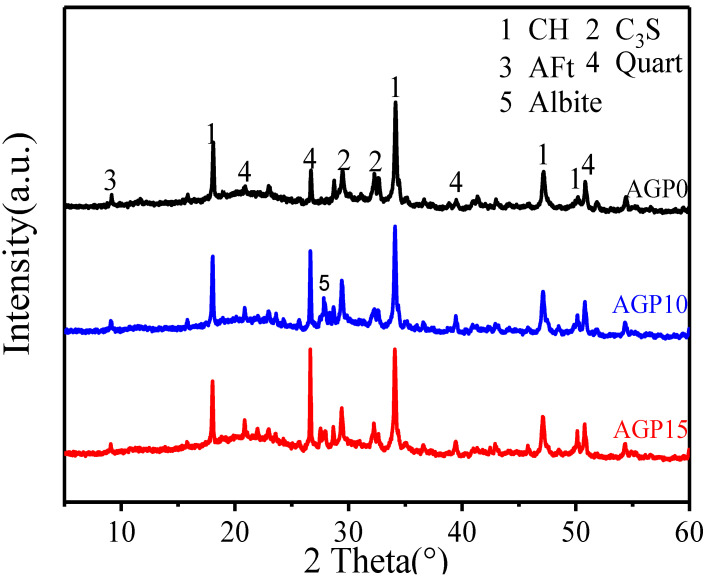
XRD patterns of 28 d samples.

The XRD results of the 28 d hydration products of granite powder-enhanced concrete are shown in Figure 11. Compared with the control group (AGP0), there were significantly higher proportions of quartz and albite phases in the AGP10 and AGP15 groups. The characteristic peak of calcium hydroxide (CH) in the AGP10 group is higher than that in the AGP0 group, indicating a higher degree of hydration. Due to the presence of certain nucleation in granite powder, CH crystallization is promoted. The CH characteristic peak of the AGP15 group is lower, which is due to the high content and the excessive reduction in the total amount of cement, which leads to the reduction in the amount of hydration products, and the dilution of granite powder leads to a reduction in the hydration degree.

## 4. Conclusions

Granite powder was incorporated to improve the mechanical properties of mass-manufactured sand concrete. The mechanical strength of mass machine-made sand concrete increases first and then decreases with the increase in granite powder. The 28 d compressive strength with the 5% and 10% replacements increased by 17.6% and 8.8%, and the 28 d flexural strength increased by 20.9% and 10.3%, respectively. Granite powder with an appropriate replacement can reduce the autogenous shrinkage. Compared with the control group, the autogenous shrinkage rate of the concrete with the 10% granite powder replacement decreased significantly by 19.7%. The heat of hydration of the cementitious material was reduced by incorporating the granite powder. The porosity was decreased significantly within a certain amount of granite powder. The granite powder did not change the type of the hydration products but played a role in the microcrystalline nucleation during the hydration process.

## Figures and Tables

**Table 1:** Chemical compositions of cement and granite powder (%).

Items	CaO	SiO_2_	Al_2_O_3_	Fe_2_O_3_	MgO	K_2_O	Na_2_O
Cement	59.3	21.28	5.99	3.31	2.16	0.13	0.49
Granite powder	2.18	70.36	14.56	2.41	1.22	5.37	2.51

**Table 2:** Sieve analysis of fine aggregate (passing, %).

Sieve Opening (mm)	9.5	4.75	2.36	1.18	0.6	0.3	0.15	0.075	Fineness Modulus
MS	100	95.1	65.2	49.8	31.2	14.2	8.2	3.6	3.28
FS	100	100	100	100	98.1	54.0	12.6	1.3	1.35

**Table 3:** Concrete mixture proportions (kg/m^3^).

Items	Cementitious Materials			Coarse Aggregate	Fine Aggregate	Water	Superplasticizer
	Cement	Granite Powder	Fly Ash				
AGP0	338.40	0	59.4	1056	704	151.2	0.75
AGP5	321.48	16.92	59.4	1056	704	151.2	0.75
AGP10	304.56	33.84	59.4	1056	704	151.2	0.75
AGP15	287.64	50.76	59.4	1056	704	151.2	0.75

**Table 4:** Cumulative heat release of cement at 150 h.

Mixture	Hydration Heat (J/g)	Relative Increase (%)
AGP0	250.98	0
AGP5	245.88	−2.03
AGP10	240.38	−4.22
AGP15	232.84	−7.23

**Table 5:** Test results of concrete pore structure.

Mixture	Total Pore Volume (mL/g)	Total Hole Area (m^2^/g)	Average Pore Size (nm)	Porosity (%)
AGP0	0.0535	6.009	15.35	11.9
AGP5	0.055	8.623	21.51	9.58
AGP10	0.036	7.734	15.68	6.50
AGP15	0.0694	13.74	19.51	11.9

## Data Availability

The original contributions presented in the study are included in the article, further inquiries can be directed to the corresponding author.
